# Circulatory factors associated with function and prognosis in patients with severe heart failure

**DOI:** 10.1007/s00392-019-01554-3

**Published:** 2019-09-27

**Authors:** Eric Rullman, Michael Melin, Mirko Mandić, Adrian Gonon, Rodrigo Fernandez-Gonzalo, Thomas Gustafsson

**Affiliations:** Department of Laboratory Medicine, Division of Clinical Physiology, Karolinska Institutet, and Unit of Clinical Physiology, Karolinska University Hospital, Stockholm, Sweden

**Keywords:** Principal component analysis (PCA), New york heart association (NYHA) functional classification, Multiplex immunoassay, Orthogonal projections to latent structures discriminant analysis (OPLS-DA)

## Abstract

**Background:**

Multiple circulatory factors are increased in heart failure (HF). Many have been linked to cardiac and/or skeletal muscle tissue processes, which in turn might influence physical activity and/or capacity during HF. This study aimed to provide a better understanding of the mechanisms linking HF with the loss of peripheral function.

**Methods and results:**

Physical capacity measured by maximum oxygen uptake, myocardial function (measured by echocardiography), physical activity (measured by accelerometry), and mortality data was collected for patients with severe symptomatic heart failure an ejection fraction < 35% (*n* = 66) and controls (*n* = 28). Plasma circulatory factors were quantified using a multiplex immunoassay. Multivariate (orthogonal projections to latent structures discriminant analysis) and univariate analyses identified many factors that differed significantly between HF and control subjects, mainly involving biological functions related to cell growth and cell adhesion, extracellular matrix organization, angiogenesis, and inflammation. Then, using principal component analysis, links between circulatory factors and physical capacity, daily physical activity, and myocardial function were identified. A subset of ten biomarkers differentially expressed in patients with HF vs controls covaried with physical capacity, daily physical activity, and myocardial function; eight of these also carried prognostic value. These included established plasma biomarkers of HF, such as NT-proBNP and ST2 along with recently identified factors such as GDF15, IGFBP7, and TfR, as well as a new factor, galectin-4.

**Conclusions:**

These findings reinforce the importance of systemic circulatory factors linked to hemodynamic stress responses and inflammation in the pathogenesis and progress of HF disease. They also support established biomarkers for HF and suggest new plausible markers.

**Graphic abstract:**

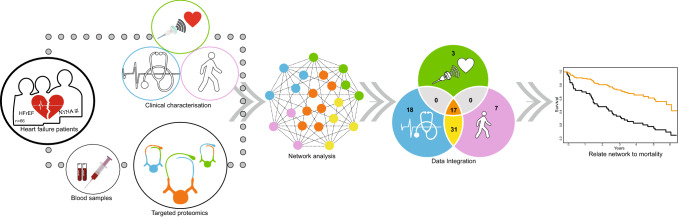

**Electronic supplementary material:**

The online version of this article (10.1007/s00392-019-01554-3) contains supplementary material, which is available to authorized users.

## Introduction

Heart failure (HF) is manifested by an inability of the heart to supply the peripheral tissues with the necessary volume of blood to meet their metabolic demands [73]. Limitation of exercise capacity because of dyspnea and fatigue is one of the cardinal manifestations of HF, increasing in parallel with the severity of the disease [16, 57, 72]. Indeed, symptoms linked to physical fatigue are among the most important factors underlying the progressive deterioration of quality of life in patients with HF [61], and decreased maximal exercise capacity is strongly associated with reduced patient survival [23, 54]. Thus, although HF is usually diagnosed by echocardiography assessment of systolic and diastolic cardiac function [65, 85], the prognostic value of this measurement is considerably smaller than peak oxygen uptake (*V*O_2peak_) [5, 23]. *V*O_2peak_ is a measure that provides an indirect assessment of a patient’s ability to increase both cardiac output and skeletal muscle oxygen uptake and represents the physical capacity of an individual.

One strategy to gain more information about the prognosis of HF consists in the generation of models that consider multiple clinically relevant variables. Thus, the combination of physical capacity (*V*O_2peak_), systolic function expressed as the left ventricular ejection fraction (LVEF), blood pressure, and heart rate has repeatedly shown the ability to identify patients with a poor prognosis [1, 48, 64]. Currently, these variables constitute the cornerstone of several well-established prognostic models for patients with HF. Nevertheless, there is a constant need for the refinement of such prognostic models, and the inclusion of new variables may help to increase their validity. Thus, the assessment of daily physical activity has gained attention in recent years [22, 25, 43]. For example, we showed that the amount of physical activity (measured by accelerometry) and the quality of such activity (i.e., intensity) were independent predictors of mortality in patients with chronic HF [56]. The rationale behind the belief that models for predicting HF will benefit from the inclusion of daily physical activity lies in the progression of the disease per se; thus, as cardiac contractile and chronotropic functions deteriorate, the patient’s capacity for daily physical activity also decreases.

To counteract failing cardiac function, numerous processes are activated aiming to maintain central hemodynamics [34]. In particular, the sympathetic nervous and renin–angiotensin systems have received considerable attention in terms of the pathophysiology and progression of the disease. However, a large number of other circulatory factors such as natriuretic peptides and cytokines have also been shown to increase in cases of HF [14]. Many of these factors are related to physical capacity, and display biological function(s) that might impact on cardiac and/or skeletal muscle tissues [4, 18, 25, 29, 38, 41, 42, 47, 60, 71]. Thus, physical activity, synergistically with increases in neuroendocrine factors, is critical to connect the reduced myocardial function of patients with HF with the alterations in peripheral tissues (e.g., kidney and skeletal muscle) associated with this disease. With this in mind, expanding our knowledge of circulatory factors in relation to HF could provide new information about the pathophysiology of the disease, allowing for analysis of the cross-talk that takes place between multiple organs of the body that are affected (e.g., heart, kidney, and skeletal muscle). In addition, exploring the relationship between such circulatory factors and the functional alterations triggered by HF (e.g., physical capacity, physical activity, myocardial function, and mortality) could offer new paths for clinical interventions targeting the disease at multiple levels/organs simultaneously.

Here, we explored the relationships between myocardial function (assessed by echocardiography, heart rate, and blood pressure measurements), physical capacity (*V*O_2peak_), daily physical activity (assessed by accelerometry), circulatory biomarkers, and cardiovascular mortality over 5 years in patients with severe heart failure with reduced ejection fraction. The general aim of this study was to provide a better understanding of the mechanisms that link the failing heart with the loss of peripheral function. More specifically, we aimed to identify circulatory factors that differed between patients with HF and controls and, using a principal component analysis (PCA) approach, were associated with established physiological prognostics variables (i.e., myocardial function, physical capacity, and/or daily physical activity) and mortality.

## Methods

### Patient population and study design

Patients were enrolled prospectively from the outpatient clinic at Karolinska University Hospital (Stockholm, Sweden). Patients with moderate to severe and stable chronic HF, defined as functional New York Heart Association (NYHA) class III disease, with no acute hospital admission within the last 8 weeks and with a left ventricular ejection fraction (LVEF) < 35% were eligible for inclusion. NYHA III was defined as a self-reported maximum continuous walking distance of no more than 200 m with dyspnea as a limiting factor. Patients fulfilling the inclusion criteria were invited to participate during scheduled visits to the attending cardiologist. After receiving written and verbal information about the study design, objectives, and potential risks, all participants signed a written informed consent form. Sixty-six patients were recruited between May 2009 and June 2013. To access a group of control subjects as closely matched as possible to patients with HF in terms of age, comorbidities, and physical activity, control subjects were enrolled among patients admitted to the outpatient clinic with symptoms suggestive of HF, but where the presence of HF was ruled out based on LVEF > 50% and a level of N-terminal pro-brain natriuretic peptide (NT-proBNP) < 300 ng/L. Echocardiographic signs of diastolic dysfunction were not an exclusion criterion in the control group, as long as the NT-proBNP level was within the normal range. The validity of the study group was checked by comparing baseline characteristics of the study group with the underlying cohort of outpatients enlisted at the hospital. The study was approved by the Regional Ethical Review Board in Stockholm, Sweden (no. 2007/1410-31/3) and carried out in accordance with the International Code of Medical Ethics of the World Medical Association (Declaration of Helsinki, 5th revision).

### Clinical examination

All participants (patients and controls) underwent echocardiography, and measurements of mean arterial pressure (MAP) and heart rate (HR) at rest (Table 1). Blood samples were collected with the subject in a fasting state in the morning in EDTA-coated tubes, and then centrifuged; plasma was aliquoted and stored at − 70 °C until analysis (Table 1). All subjects performed a symptom-limited cardiopulmonary exercise test (CPX) to assess *V*O_2peak_ (Table 1). The CPX consisted of maximum symptom-limited exercise either on a cycle ergometer (increments of 10 W every 60 s) or on a treadmill (1 m/s with a stepwise increase in the angle of 0.5°/min). In every CPX performed, continuous assessment of gas-exchange data (Vmax, SensorMedics, Anaheim, CA, USA) was performed. The exercise was terminated due to volitional exhaustion and/or the patient’s inability to maintain the speed of 1 m/s (treadmill) or a cadence of 60 rpm (cycle ergometer) despite strong verbal encouragement. Echocardiographic measurements were carried out in accordance with clinical guidelines (Vivid 7, General Electrics Healthcare, Little Chalfont, United Kingdom) and was analyzed by an echocardiographer blinded to the specific clinical history of the patient. Left ventricular end-diastolic diameter (LVEDD) was measured and left ventricular ejection fraction (LVEF) was calculated using the biplane Simpson's rule. Diastolic function was estimated based on mitral inflow deceleration time (DT) and for patients with sinus-rhythm mitral in flow E/A-wave. Daily activity was assessed in all participants by accelerometers (GT3X; Actigraph, Pensacola, FL, USA), which were mailed to all patients within 6 weeks of the CPX measurements. The patients were instructed to attach the accelerometer to their waist belt upon rising in the morning and to remove it only for showering, bathing, and sleeping. The monitors were set to begin collecting data 1 day before the delivery date, as estimated by the postal service, and to continue recording data until they were downloaded. The patients were asked to return the monitor by mail using a prepaid return envelope after having worn it for seven consecutive days. Raw data collected by the accelerometer were integrated into 60-s epochs using ActiLife software with the normal filter option and expressed as counts per minutes (cpm). Wear time was estimated using the algorithm described by Troiano et al. [78]. Nonwear time was defined as 60 consecutive minutes of 0 cpm, with allowance for 1–2 min of 0–99 cpm during this time. Patients with an estimated wear time of < 3 days were eliminated from further analysis (*n* = 3). In addition to analysis of time spent active, skewness was analyzed as a potentially important prognostic measure of variance in the level of physical activity [56]. Data on mortality and cause of death were obtained from the Swedish national cause-of-death registry in June 2015.

**Table 1:** Continuous variables are presented as median and lower and upper quartiles (Q1; Q3) and categorical variables as numbers (*n*) and percentages

Baseline characteristics	Heart failure (*n* = 66)	Controls (*n* = 28)	*p* value
Demographics			
Age, years	70 (63; 74)	70.5 (65; 72.2)	n.s.
Female	13 (20)	20 (71)	< 0.0001
BMI	27.5 (25; 30.2)	25.5 (23; 29.8)	n.s.
SBP, mmHg	117.5 (108.8; 135)	150 (140; 160)	< 0.0001
DBP, mmHg	75 (60; 80)	85 (80; 90)	< 0.0001
NYHA functional class			
III	63 (95)	–	
IV	3 (5)	–	
Heart rate, bpm	72.5 (64.8; 80)	72 (67; 76)	n.s.
Peak *V*O_2_, ml/(kg min)	13.4 (12; 16.1)	23.8 (17.7; 25.4)	< 0.0001
Comorbidities			
Diabetes mellitus	29 (44)	3 (11)	< 0.005
COPD	11 (17)	7 (25)	< 0.05
Hypertension	38 (58)	18 (64)	n.s.
Atrial fibrillation	36 (55)	1 (4)	< 0.0001
Clinical chemistry			
NT-proBNP, ng/l	2210 (1070; 5410)	107 (61; 273.5)	< 0.0001
Creatinine clearance, ml/min	58 (44; 86)	75 (59.5; 84.5)	n.s.
Hemoglobin, g/dl	141 (127; 154)	135.5 (131; 142.5)	n.s.
Medication			
ACEi	43 (65)	5 (18)	< 0.0001
ARB	22 (33)	7 (25)	n.s.
β-Blockers	63 (95)	8 (7)	< 0.0001
MRA	38 (58)	0 (0)	< 0.0001
Diuretic agents	62 (94)	8 (29)	< 0.0001
Echocardiographic measurements			
LVEF, %	25 (20; 32)	57.75 (55; 60)	< 0.0001
LVEDD, mm	64 (59; 71.2)	46 (42; 52)	< 0.0001
PSV, cm/s	0.04 (0.03; 0.05)	0.07 (0.06; 0.08)	< 0.0001
LVOT, m/s	0.7 (0.6; 0.9)	0.9 (0.9; 1)	< 0.0001
Septal *É*, cm/s	0.04 (0.04; 0.05)	0.07 (0.06; 0.08)	< 0.0001
Lateral *É*, cm/s	0.05 (0.04; 0.08)	0.08 (0.07; 0.11)	< 0.0001
*E*/*É*	16.4 (12.7; 24)	9.5 (7.6; 11.8)	< 0.0001
LA-area, cm^2^	31 (26; 36)	19 (15.2; 20.8)	< 0.0001
PA-pressure, mmHg	47 (40; 52.9)	30 (30; 40)	< 0.0001
TAPSE, mm	15 (11.5; 18)	23 (18; 25)	< 0.0001
Daily physical activity			
Time spent active, %	22 (17;30)	38 (31;44)	< 0.0001
Time spent inactive, %	78 (70; 83)	62 (56;69)	< 0.0001
Skewness, cpm	1.6 (0.8;2.5)	1.0 (0.8;1.5)	< 0.05

Continuous variables were tested using *t* test and frequencies using Chi-square test

*ACEi* angiotensin converting enzyme inhibitor, *ARB* angiotensin receptor blocker, *β-blockers* beta blockers, *BMI* body mass index, *COPD* chronic obstructive pulmonary disease, *CRT* cardiac resynchronization therapy, *DM* diabetes mellitus, *ICD* implantable cardioverter defibrillator, *IHD* ischemic heart disease, *LVEF* left ventricle ejection fraction, *MRA* mineralreceptorantagonist, *n.s.* non-significant, *NT-proBNP* NT-pro-brain natriuretic peptide, *NYHA* New York Heart Association

### Measurement of circulating factors

Factors were quantified from single samples using a multiplex immunoassay (Proseek Multiplex 96 × 96 CVD III; Olink Bioscience, Uppsala, Sweden), which is a 92-plex immunoassay based on a proximity ligation extension assay. Proximity extension assays use target-specific antibody pairs that are linked to DNA strands that, upon simultaneous binding to the target analyte, create a real-time polymerase chain reaction amplicon in a proximity-dependent manner enabled by the action of a DNA polymerase. The intra-assay coefficient of variation (CV) ranges between 5 and 11% (mean 6%), and the inter-assay CV ranges between 9 and 39% (mean 15%) [8]. All factors in the assay have been validated and relevant spike-in experiments have been performed to ensure that there is no cross-reactivity between the different biomarkers [51]. Further information about reproducibility and validation can be found at https://www.olink.com. NT-proBNP levels were analyzed by electrochemiluminescence immunoassay using cobase immunoassay analyzers (Roche Diagnostics, Rotkreuz, Switzerland). Creatinine clearance was calculated according to the Cockcroft–Gault formula.

### Data analysis

Descriptive data are expressed as medians and quartiles (Q1–Q3), or as numbers and (%). The concentrations of the biomarkers are expressed in arbitrary units. Univariate groupwise comparisons were carried out by two-sided Student’s *t*, Chi-squared, and Fisher’s exact tests as appropriate. As an additional control for our measurements, Bland–Altman analysis was used to compare values of NT-proBNP measured by the Proseek platform and an immunofluorescence assay.

### Principal components analysis (PCA)

Given the large set of variables, the reduction of the dimension of our covariate list was performed through variable clustering and dimensionality reduction through PCA Bi plots was used to characterize the variance of the individual variables and to identify collinearities. Variables derived from echocardiographic assessment [LVEF, left ventricular end-diastolic diameter (LVEDD), peak systolic velocity, septal, and lateral *E*′ values, LA-area, estimated PA-pressure, and tricuspid annular plane systolic excursion (TAPSE)], clinical examination and exercise test (MAP, heart rate, and *V*O_2peak_), and daily physical activity (time spent active, time spent inactive, and skewness) were analyzed. All variables were scaled to unit variance and mean-centered. The concentrations of the Proseek biomarkers are expressed as arbitrary units and were mean-centered prior to analysis because of the lack of specific calibrators for the assays. Principal components were calculated using the ‘prcomp’ function in the R statistics package (R Foundation for Statistical Computing, Vienna, Austria), and biplots were generated using the ‘factominer’ and ‘ggplot2’ libraries. Ontological enrichment analysis was performed using the web-based Database for Annotation, Visualization, and Integrated Discovery (DAVID).

### Orthogonal projections to latent structures discriminant analysis (OPLS-DA)

For the case–control analysis, we used OPLS, which is similar to PCA, but was developed to handle classification rather than correlation. OPLS regression is particularly suited when the matrix of predictors has more variables than observations and when there is multicollinearity among *X* values [77]. An OPLS-DA classification model was constructed using the rOPLS-library in R [74], in which the contribution of each variable is represented by a loading value compared with what is predicted, in this case, patient or control. An OPLS model finds the multidimensional direction in the *X* space that explains the maximal variance in the *Y* space. The validity of the model (*Q*_2_ value) was assessed by bootstrapped cross validation, yielding 95% confidence intervals for the contribution of each of the variables in the group classification.

### Network inference

Given the high covariance of the physiological measures, the dimensions of our covariate list were reduced utilizing summarized measures of each category derived from the principal components [79]. This approach retains variance/information that is shared between several of the variables and discards less systematic variance that is more likely to contain noise [84]. This process results in a summary score for each category, as a linear combination of variables within the category summarized for each patient. The two first principal components were kept from each of the categories, physical capacity, daily physical activity, and myocardial function. To develop networks representing the interaction between plasma factors and HF pathophysiology, a global interaction network was constructed using ARACNE, an algorithm for the reconstruction of gene regulatory networks in a mammalian cellular context. This is a validated network inference technique that infers the interaction between pairs of variables using a measure of correlation called mutual information (MI) [45]. MI between each pair of variables was tested using a permutation-based statistical test. ARACNE offers numerous advantages over more traditional measures of correlation, including the ability to spot nonlinear correlations, which are very effective in identifying biologically relevant connections [26]. Significant connections were identified by applying a *P* value threshold for significant MI values corresponding to a false discovery rate of 5%. To avoid potential overfitting in the ARACNE network, a correlation matrix was constructed, with confidence intervals estimated for each pairwise correlation through bootstrapping. Only pairwise correlations with an absolute correlation of > 0.2 and a false discovery rate (FDR) of < 5% were retained. The resulting network was visualized using the software application Cytoscape v. 3.5 (https://www.cytoscape.org) [70].

### Cox regression analysis of mortality

Associations with the outcome were determined with Cox proportional hazards models and presented as hazard ratios (HRs) and 95% confidence intervals and with two-sided *P* values. In the final multivariable Cox regression model, three clinically significant covariates, age, sex, and NT-proBNP concentrations, were included together with each biomarker. All biomarkers were analyzed in log2-transformed format using the survival package on the statistical platform R version 3.5.0.

## Results

### Clinical data and mortality rates

Sixty-six patients with HF and 28 controls were enrolled in the study over a period of 3 years. The demographic and clinical characteristics of the subjects included are presented in Table 1. Within the HF patient cohort, *V*O_2peak_ was on average 13.4 mL/kg (range 6.1–22.5 mL/kg), and 23.8 mL/kg/min (17.7–25.4 mL/kg) for controls. Among the patients, time spent physically active (>100 counts/min) was 950 (IQR 541–1366) min/week, and 2182 (IQR 1592–2790) min/week for controls. The follow-up time for surviving patients with HF was 3 years (range 1–5). Of the 66 monitored patients, 42 died during follow-up (median time 1.8 years). All mortality events were categorized as cardiovascular in nature. To confirm validity of the study group, baseline characteristics of the patients were compared with data on all patients enlisted as heart failure outpatients at the hospital at the time of enrollment (*n* = 1467). The patients enrolled in the study where similar to the average patient with regards to age, body composition, comorbidities, and underlying conditions but hade worse cardiac function and functional status, fewer females and had more aggressive medical treatment than the average patient (Supplemental Table).

### Characteristics of plasma factors

**Fig. 1:**
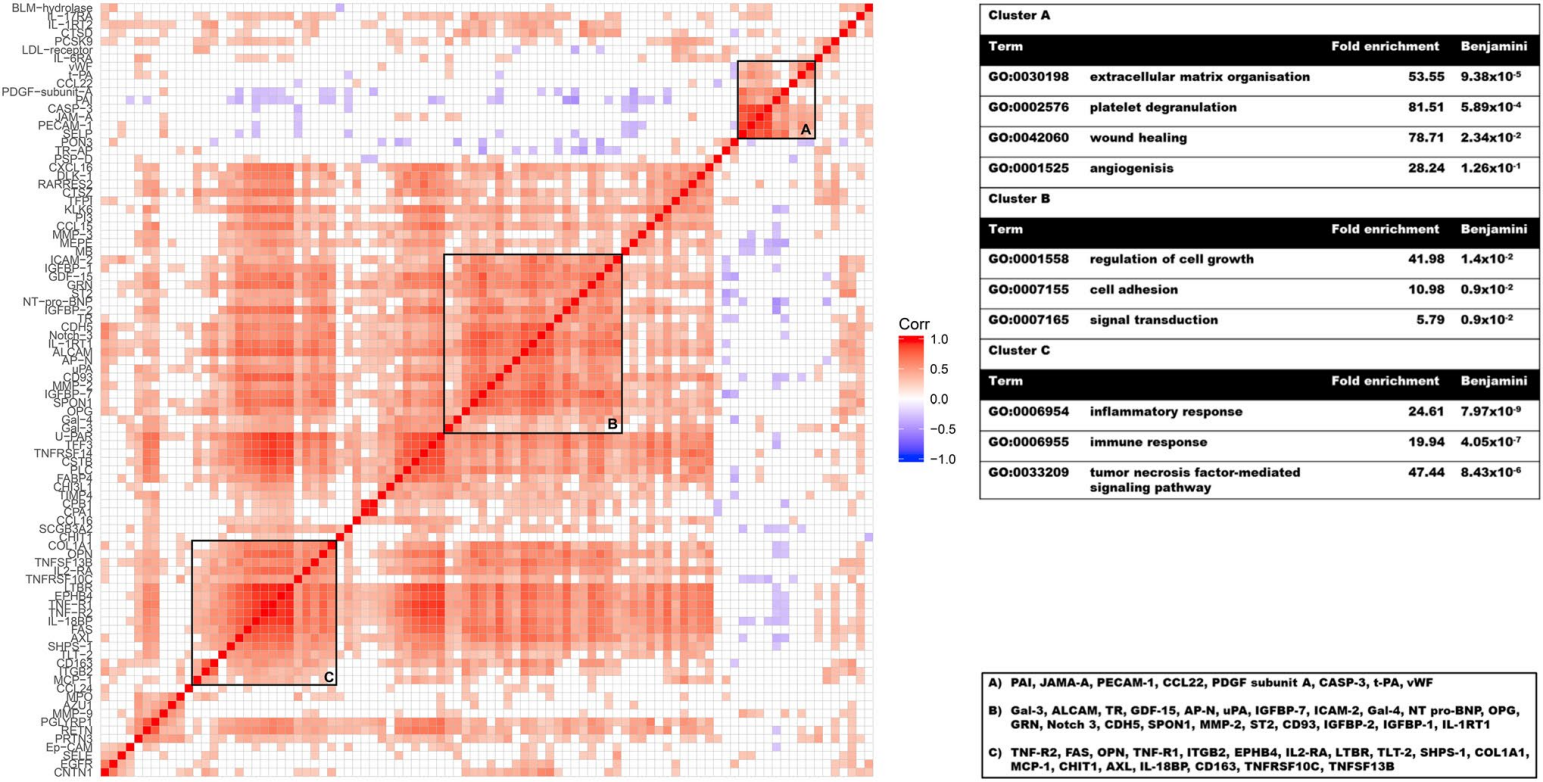
Correlation matrix illustrating mutual correlation amongst all biomarkers. Red and blue denote statistically significant positive and negative correlations, respectively. A large number of biomarkers are highly positively correlated and visual inspection indicates that there are clusters of variables with very high degree of correlation apparent, shown as bright red squares on the correlation matrix

There was a high overall correlation across the different factors with a mean Pearson correlation coefficient of *R*^2^ = 0.25. For these, PCA captured ~ 48% of the total variance explained by the first three principal components. Many of the factors assessed in the Proseek assay belong to the same family (e.g., tumor necrosis factor [TNF]-alpha, leukocyte adhesion molecules) or share similar functions. Indeed, ontological enrichment analysis showed that the assay as a whole is highly enriched for the cellular factors inflammation, cellular adhesion, and migration (Fig. 1). Despite this innate bias, clusters of highly correlated factors were tested for functional enrichment. A correlation matrix was constructed, and cluster analysis was performed based on pairwise correlations between variables. This revealed that the measured factors formed clearly distinguishable clusters with a high degree of correlation across observations, in which factors with similar biological functions cluster more closely together. That factors correlate according to their biological functions serves as a biological quality control; if data contained mainly noise or were caused by methodological bias, the correlations would have been random and/or unrelated to biological functions. Ontological enrichment analysis using DAVID identified three distinguishable clusters with different biological functions (Fig. 1). The first cluster was highly overrepresented by factors with described biological functions related to the regulation of cell growth and cell adhesion. The second cluster was most enriched for extracellular matrix organization factors and angiogenesis. The third cluster was enriched for inflammatory response markers. As an additional control for our measurements, NT-proBNP was analyzed using an immunofluorescence assay, in addition to the measurements performed using the Proseek platform. The agreement between the two methods was “very good”, with an *R*^2^ correlation coefficient of 0.91, and a difference within 0.5 standard deviations for all observations shown by Bland–Altman analysis.

### Case–control analysis

**Fig. 2:**
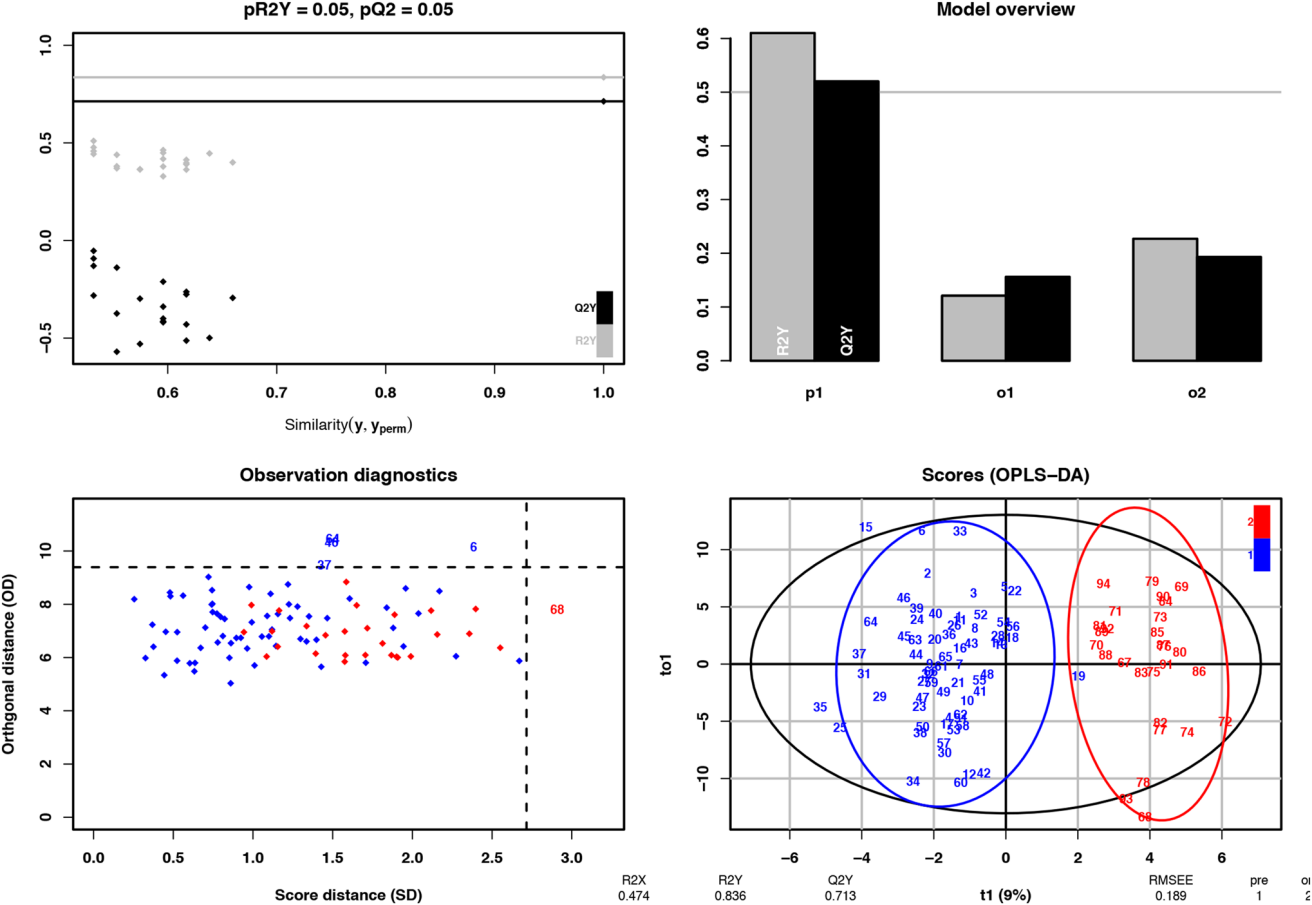
For the case–control analysis, we used OPLS which is similar to PCA, but developed to handle classification rather than correlation: an OPLS model will try to find the multidimensional direction in the *X* space that explains the maximum multidimensional variance direction in the *Y* space. OPLS regression is particularly suited when the matrix of predictors has more variables than observations and when there is multicolinearity among *X* values. The OPLS model differentiating patients form controls class correctly classified 84% (*R*^2^*Y* = 0.84) of the observations in the data set, with a predictive (*Q*_2_ value) of 0.71 after cross validation

Based on the high covariance among the investigated circulatory factors, the differences in the concentrations analyzed in plasma between patients with HF and controls were explored through a multivariate approach. The OPLS model differentiating patients from controls correctly classified 84% (*R*^2^*Y* = 0.84) of the observations in the data set, with a predictive (*Q*_2_ value) of 0.71 after cross validation (Fig. 2). The model identified 39 factors that contributed significantly to the model (18 were higher and 21 were lower in patients vs controls, respectively). The results are summarized in Table 2, together with the corresponding parametrically tested FDRs. As implied in the original methodology paper [74], there was a strong correlation between Variable Importance in Projection from the OPLS-DA classifier and *P* values based on parametric testing. A VIP threshold from the OPLS-DA of 0.8 corresponds to a parametric FDR of 6% (Table 2).

**Table 2:** Factors significantly different between patients and controls

Name		Uniprot	Ratio	FDR	VIP
EGFR	Epidermal growth factor receptor	P00533	0.3	< 0.001	2.6
NT pro-BNP	N-terminal prohormone brain natriuretic peptide		569	< 0.001	2.5
PON3	Paraoxonase (PON3)	Q15166	0.1	< 0.001	2.6
TLT-2	Trem-like transcript 2 protein	Q5T2D2	0.2	< 0.001	1.9
TFPI	Tissue factor pathway inhibitor	P10646	0.3	< 0.001	1.9
GDF-15	Growth/differentiation factor 15	Q99988	10.3	< 0.001	2.0
PAI	Plasminogen activator inhibitor 1	P05121	0.1	< 0.001	1.2
U-PAR	Urokinase plasminogen activator surface receptor	Q03405	3.2	< 0.001	1.5
MMP-3	Matrix metalloproteinase-3	P08254	4	0.001	1.5
SELP	P-selectin	P16109	0.2	0.001	1.2
FABP4	Fatty acid-binding protein, adipocyte	P15090	10.2	0.001	1.5
CNTN1	Contactin-1	Q12860	0.5	0.001	1.5
TR-AP	Tartrate-resistant acid phosphatase type 5	P13686	0.4	0.002	1.7
PDGF subunit-A	Platelet-derived growth factor subunit A	P04085	0.1	0.002	1.2
SPON1	Spondin-1	Q9HCB6	2.6	0.002	1.3
DLK-1	Protein delta homolog 1	P80370	0.3	0.003	1.4
ITGB2	Integrin beta-2	P05107	0.4	0.003	1.2
PI3	Elafin	P19957	4.2	0.003	1.2
LDL-receptor	Low-density lipoprotein receptor	P01130	0.3	0.003	1.3
Gal-4	Galectin-4	P56470	2.5	0.004	1.6
ST2	ST2 protein	Q01638	3.3	0.005	1.4
COL1A1	Collagen alpha-1(I) chain	P02452	0.4	0.005	1.4
CASP-3	Caspase-3	P42574	0.1	0.006	0.9
OPN	Osteopontin	P10451	2.9	0.007	1.2
BLM hydrolase	Bleomycin hydrolase	Q13867	0.5	0.008	1.1
TFF3	Trefoil factor 3	Q07654	3.1	0.008	1.1
TR	Transferrin receptor protein 1	P02786	3.3	0.008	1.3
PLC	Perlecan	P98160	2.1	0.009	1.2
CSTB	Cystatin-B	P04080	3.2	0.010	1.1
IGFBP-7	Insulin-like growth factor-binding protein 7	Q16270	3.2	0.011	1.2
TNF-R1	Tumor necrosis factor receptor 1	P19438	2.7	0.012	1.1
PECAM-1	Platelet endothelial cell adhesion molecule	P16284	0.3	0.019	0.7
TNF-R2	Tumor necrosis factor receptor 2	P20333	2.4	0.020	0.9
FAS	Tumor necrosis factor receptor superfamily member 6	P25445	0.6	0.021	1.0
MPO	Myeloperoxidase	P05164	0.5	0.026	0.9
PSP-D	Pulmonary surfactant-associated protein D	P35247	2.8	0.026	1.0
Ep-CAM	Epithelial cell adhesion molecule	P16422	0.3	0.030	0.9
CCL24	C–C motif chemokine 24	O00175	0.3	0.037	1.0
RARRES2	Retinoic acid receptor responder protein 2	Q99969	0.6	0.037	0.9

*Uniprot* uniprot accession, *Ratio* The ration between patients vs controls. *FDR* false discovery rate for parametric groupwise comparison. *VIP* variable importance in projection from OPLA-DA classification

### Characteristics of physiological variables

**Fig. 3:**
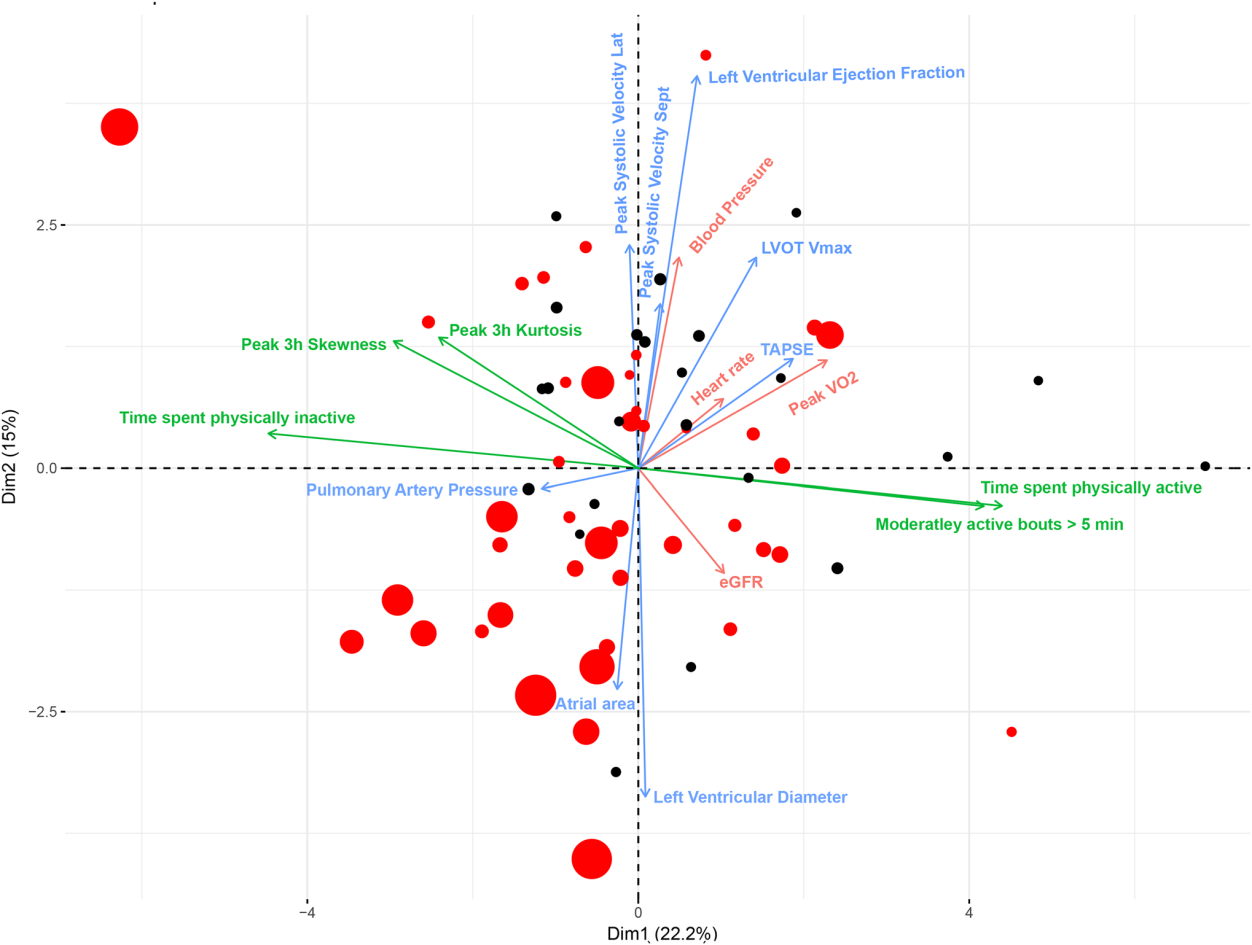
PCA and biplot on physiological variables and individual observations, where red dots denote patients suffering an event and the size of the dot is proportional to the event, larger dots denote earlier events. There was a high overall correlation between all variables and 40% of the overall variance could be captured with the first two principal components. Variables related to daily physical activity had highest loading on the 1st component and echocardiographic variables on the second. Exercise capacity and heart rate contributed to both PC1 and PC2. The biplot indicates, as expected, the prognostic utility of the investigated physiological variables: Cox proportional hazard-ratio calculated on high vs low loading on PC1 and PC2 showed an HR of 1.8 and 2.8, respectively. With both components combined the hazard ratio (HR) for patients in lower left quadrant of the PCA was 4.0 (highest risk) compared with patients in upper right quadrant (lowest risk)

The variables derived from the clinical characterization of the patients were investigated with a similar approach to that described above for factors measured in the serum. The mean Pearson correlation coefficient across variables was 0.25, and PCA on all physiological variables combined showed high overall correlation between all measured variables; 75% of the overall variance could be captured with the first three principal components. Thus, while keeping most of the information intact, it was possible to summarize the clinical examination data into three principal components. This scaled down the number of dimensions and considerably reduced the background noise. The variables with the highest loading on principal component 1 were *V*O_2peak_, MAP, heart rate, and daily physical activity; on principal component 2, the measures of both systolic (LVEF and peak systolic velocity) and diastolic dysfunction (*E*′, estimated PA-pressure, E-max and LA-area) had the highest loading. Physical capacity and heart rate contributed to both principal components 1 and 2, illustrating that they share some variance with both daily physical activity and measures of systolic function (Fig. 3). To validate and visualize prognostic information, a bi-plot was constructed in which data on mortality and time to mortality events were added to the PCA (Fig. 3) and analyzed with a Cox regression model. As expected, the prognostic utility of the investigated physiological variables was retained when summarized on PCA.

### Associations between plasma factors and pathophysiological changes

**Fig. 4:**
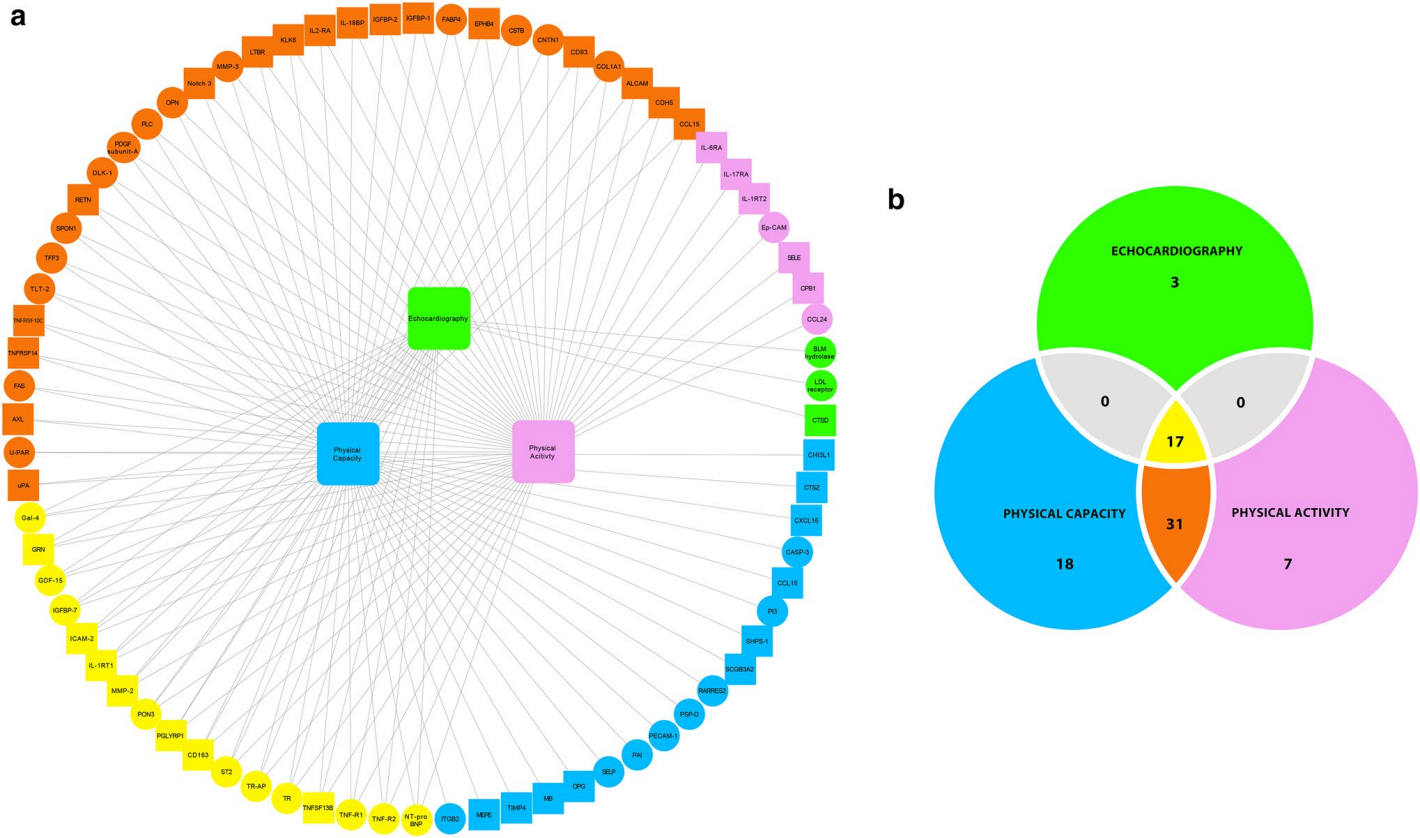
Network inference edges **a** denote significant correlations and the length of each edge is inversely proportional to the strength of the correlation. Thus, nodes appearing closely together share higher number of significant edges, and large nodes indicate key markers with many significant connections. The network analysis identified 17 biomarkers, **b** relating to the majority of the clinical components and one, GDF15 had significant connections to all components. These key markers contained both classical biomarkers considered to reflect cardiac stretch (ST2 and BNP) but also a large number of inflammatory components and factors related to metabolism such as IGFBP7

To identify associations between plasma factors and pathophysiological changes in HF, the circulating factors in the patients with HF were associated with the measured physiological variables with MI network inference, and covariance was calculated; that is, how large a portion of the variance in one variable can be accounted for from the variance of a second variable. The resulting network contained 76 unique factors with 141 significant edges relating the investigated physiological categories of variables (Fig. 4a). Of the physiological categories, physical capacity and daily physical activity were the most centrally placed hubs, and 48 of the 76 factors were connected to both physical capacity and daily physical activity. Apart from having the largest number of significant connections, physical capacity also had the highest number of connections to unique factors (Fig. 4b). The components derived from echocardiography, such as myocardial function, had fewer connections to factors, with only 20 significant edges, and these were shared with the other physiological categories (Fig. 4b). Of these 20 factors, 10 were among the 39 factors that differed significantly between patients and controls (Tables 2, 3). Some of these factors are considered to reflect cardiac stretching (ST2 and NT-proBNP), and others are linked to inflammatory responses (members of the TNF-alpha family; TNF-R2 and TNF-R1). The network analysis also identified some relatively or completely novel factors in the field of HF: growth differentiation factor 15 (GDF15), insulin-like growth factor-binding protein 7 (IGFBP7), transferrin receptor protein 1 (TfR1), and galactin-4. However, GDF15 and IGFBP7 have already been reported as potentially important markers of disease severity in HF, and are induced by hemodynamic and inflammatory stress [6, 30].

**Table 3:** Hazard ratio (HR) per quartile increase in each protein, raw *p* values (*p*) and false discovery rate (FDR) from univariate cox-regression (left) and multiple regression controlling for age, estimated Glomerular Filtration Rate (eGFR), peakVO2, and left ventricular ejection fraction (LVEF)

Biomarker	Univariate cox-regression crude analysis			Cox-regression controlled for age, eGFR, peakVO2 and LVEF		
	HR	*p*	FDR	HR	*p*	FDR
Transferrin receptor protein 1	2.2	0.000	0.001	2.1	0.000	0.001
Growth/differentiation factor 15	2.1	0.000	0.002	2.0	0.001	0.004
Galectin-4	2.7	0.001	0.007	2.6	0.002	0.005
Insulin-like growth factor-binding protein 7	1.9	0.001	0.011	1.7	0.010	0.016
ST2 protein	2.1	0.002	0.012	2.0	0.007	0.014
Tumor necrosis factor receptor 1	2.1	0.002	0.012	1.9	0.028	0.037
N-terminal prohormone brain natriuretic peptide	1.4	0.004	0.014	1.3	0.054	0.062
Tumor necrosis factor receptor 2	2.0	0.006	0.021	1.7	0.073	0.073
Paraoxonase (PON3)	0.8	0.233	0.306			
Tartrate-resistant acid phosphatase type 5	0.7	0.353	0.427			

**Fig. 5:**
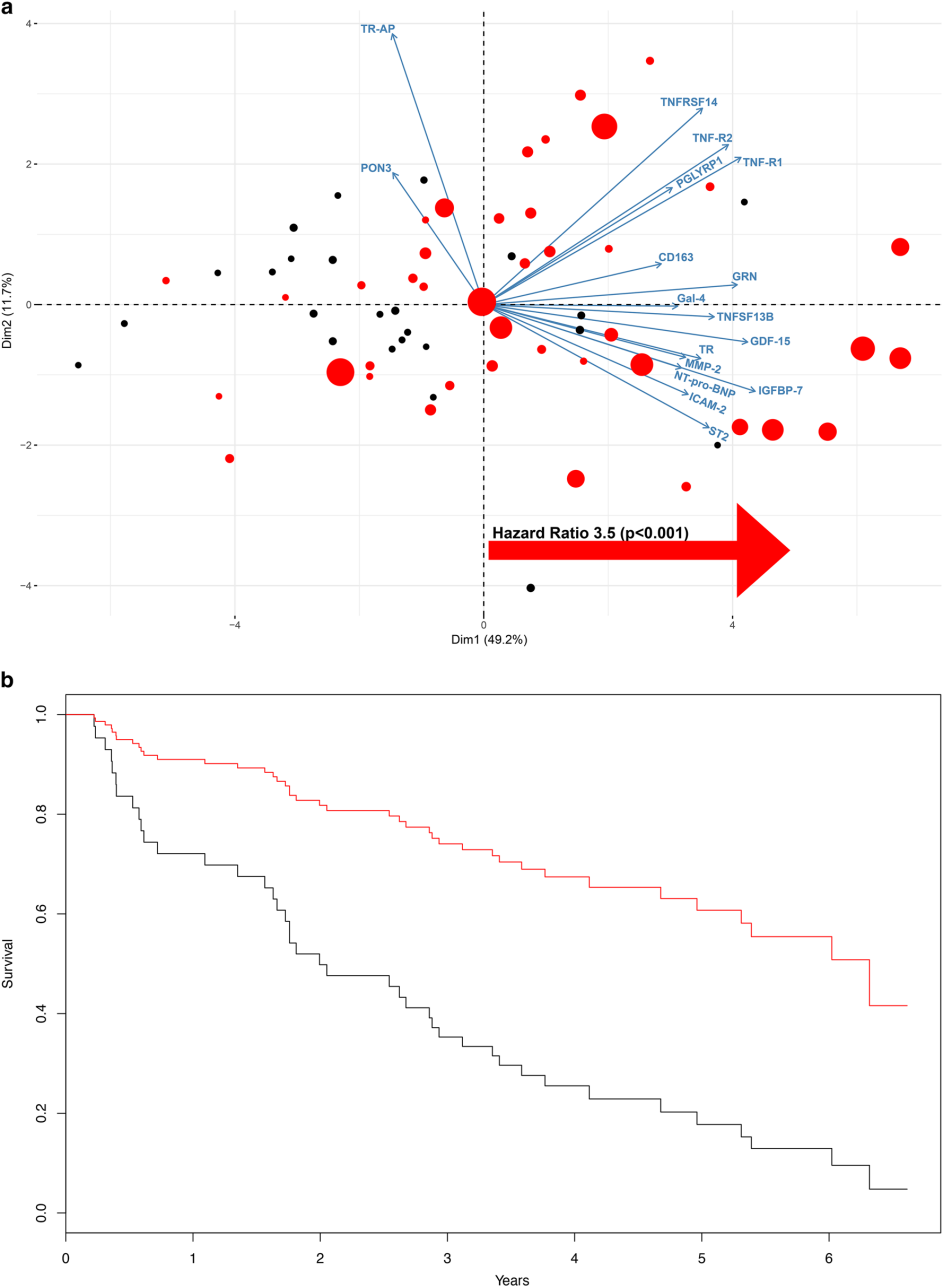
Biplot (**a**) and kaplan–meier curve (**b**) on the 16 key-network hubs vs mortality and individual observations, where red dots denote patients suffering an event and the size of the dot is proportional to the event, larger dots denote earlier event. There was a high overall correlation between all variables, and 61% of the overall variance could be captured with the first two principal components. The prognostic utility of the investigated physiological variables: Cox proportional hazard ratio calculated on high vs low loading on PC1 showed a HR of 3.5 (*p* < 0.001)

The ten factors that were expressed differently between patients with HF and controls were analyzed further to test whether they were linked with HF prognosis. This was tested by both a multivariate approach and through Cox regression. The PCA connecting physiological components to prognosis were recalculated substituting the physiological variables with the ten factors (Fig. 5a, b). As expected, because the factors were selected based on covariance and MI with prognostically important clinical variables, the combined factors reflected this prognostic value. The central network factors displayed a similar distribution of the variance as did the physiological variables (65% of variance retained within the first two components), and the HR of mortality events was 4.2 (*P* < 0.001; first component) and 1.2 [not significant (NS); second component]. In addition to this multivariate approach, the TEN network factors were also tested for prognostic value one-by-one in a Cox regression analysis, both as single independent variables and controlling for important confounders (age, estimated glomerular filtration rate [eGFR], *V*O_2peak,_ and LVEF; Table 3). All of the network factors except for PON3 and TR-AP were significantly associated with all-cause mortality (FDR < 5%) in univariate analysis (Table 3). Controlling for established clinical risk factors, TfR1, GDF15, and galectin remained significantly associated with all-cause mortality at FDR < 5% (Table 3).

## Discussion

Using a systems biology approach, we examined potential links between circulatory factors, physical capacity, physical activity, myocardial function, and mortality in patients with HF. Thirty-nine circulatory factors differed significantly between patients with HF and controls. Established prognostic markers (*V*O_2max_ and LVEF) in the patients with HF were validated with respect to their prognostic capacity in the current cohort. Of the 39 differently expressed circulating factors in the patients with HF, 34 were associated with at least one of the physiological variables measured also when analyzed in the patient group only. A subset of 17 factors covaried with myocardial function, physical capacity, and daily physical activity, of which 10 differed between patients with HF and controls. Eight of these factors ultimately contained prognostic information. This group included well-established (e.g., NT-proBNP) and more recently recognized (e.g., ST2) plasma biomarkers of HF, together with factors recently associated with this disease (e.g., GDF15, IGFBP7, TfR) and one factor that was only just now linked to HF (galectin-4) [13].

The overall aim of this study was to identify plausible circulatory factors linking reduced myocardial function to changes in peripheral tissue functions. The mutual relationships between echocardiographic measurements of myocardial function, *V*O_2peak_, heart rate, MAP, and daily physical activity confirmed their correlations with prognosis, supporting the clinical measurements as valid, as well as this HF cohort as a valid representation of the general HF with reduced ejection fraction population. The multi- (OPLS-DA) and univariate analyses identified a substantial number of factors that differed significantly between the two groups. This finding confirmed earlier hypothesis-driven ‘single-factor’ studies reporting increased circulatory levels of NT-proBNP [68, 76], ST2 [3, 7, 10, 55], GDF15 [20, 28, 32, 69, 80], and IGFBP7 [21, 30, 31, 58] in patients with HF, which have been suggested to participate in the pathogenesis and progress of HF [14]. After deconstruction of the clinical variables into three components (myocardial function, physical capacity, and physical activity), MI network analysis identified 17 circulatory factors that covaried significantly with all three components. It should be noted that such relationships do not define a direct biological connection; to identify plausible factors related to peripheral remodeling induced by the negative alterations triggered by HF, information about the biological role and the regulating stimuli for each individual factor is needed. By identifying factors linked to all the physiological categories, the outcome is also less likely to be related to noise or other confounders, because the variance between the components is shared. Thus, it is clear that the analysis strategy used here differed from proteomic explorative studies, in which proteomic hits are prioritized for validation based on differences between patients and controls, direct correlation with prognosis, or a combination of the two. A similar analytical strategy, utilizing factor–factor mutual information to construct networks of circulating biomarkers, was recently used to explore differences between heart failure with reduced (HFrEF) and preserved ejection fraction (HFpEF) [75]. Interestingly, several members of the HFrEF network were also identified as key network members in the present study. The fact the key network members are identified in several independent cohorts strengthen their validity. The current study goes one step forward by showing their relation to clinically derived prognostic variables and prognosis.

Of the 17 factors connected to all three physiological categories, 10 were distinctly higher in patients with HF than controls, and of these, eight carried prognostic information. NT-proBNP was among these. NT-proBNP has a well-documented, strong prognostic value [37], and a close relationship to cardiac function [53, 82]. Although there are reports on peripheral effects of NT-proBNP on, e.g., adipocyte lipid metabolism or peripheral fluid balance [46, 83], information about any direct effect of this factor on skeletal muscle is scarce. The association between NT-proBNP and myocardial function, and physical capacity and activity, may relate to impaired myocardial function and reduced oxygen delivery on physical activity. Yet, the potential negative effect of this factor on peripheral tissue should be addressed in more detail in future studies. ST2 is a more recently discovered, yet validated biomarker shown to have a strong link with biochemical and clinical variables in cases of HF [66]. Changes in ST2 plasma concentration have been attributed to increased cardiac stretching or filling pressures [15]. The association found here between ST2 levels, and physical capacity and physical activity, is supported by the association between ST2 and NYHA classification reported elsewhere [44]. In addition, evidence indicates that ST2 plays an important role during skeletal muscle remodeling after injury [11]. However, a distinction between ST2, and the soluble circulating form (sST2) needs to be recognize, since sST2 acts as a decoy receptor and thereby presumably with a negative impact on skeletal muscle remodeling processes [11, 33]. Two members of the TNF-alpha family (TNF-R1 and TNF-R2) were also among the circulatory factors that covaried with all three components, and these were distinctly elevated in patients with HF. The TNF-alpha family is known to correlate with functional status in cases of HF, and single-factor studies have suggested the possible connection between increased levels of TNF-alpha, and physical capacity and prognosis in such patients [38]. High levels of TNF-alpha are known to have direct negative consequences in multiple peripheral tissues. For example, in skeletal muscle, this factor hampers differentiation processes in satellite cells, and has deleterious effects on muscle metabolism, contractile function, and apoptosis [27, 49, 67].

GDF15, IGFBP7, and TfR1 are three more recently identified factors showing covariance with all three components of analysis that carry prognostic information, and were distinctly elevated in patients with HF vs controls. GDF15 appears to increase with the severity of HF disease [32], and has been suggested as a potential prognostic marker of both HFpEF and HFrEF [20, 24, 28, 32, 69]. The multivariate approach we used here not only demonstrated that GDF15 levels are elevated in patients with HF, but also underlined its covariation with a substantial number of other factors and relevant prognostic (physical) variables. GDF15 is most abundant in the liver, with lower levels in most other tissues [50]. However, GDF15 is upregulated in response to disease in multiple tissues apart from the liver, such as the myocardium [2, 36, 86], and it is also linked to inflammation and neurohormonal activation [6, 40]. Thus, taking together previous observations and our current data indicating an association of GDF15 with myocardial function, physical capacity, and activity in patients with HF, we hypothesize that GDF15 is linked to hemodynamic system stress in such patients. Especially intriguing is that many of the biological functions of GDF15 fit with the HF phenotype, from activation of hypothalamic neurons [39] leading to anorexia with drastic weight loss and cachexia [63], to direct catabolic effects on skeletal muscle [12] and a negative impact on Growth hormone-Insulin like Growth Factor-1 axis (GH-IGF1 axis) [81]. IGFBP7 is a recent discovery in the context of HF biomarkers [21], and unlike the majority of biomarkers, its discovery was based on a proteomic assay rather than a hypothesis-driven approach [14]. Previous observations indicating a relationship between IGFBP7 and diastolic function and exercise capacity [30] are in line with our findings. However, the mechanistic role for IGFBP7 in the progression of HF remains elusive. IGFBP7 is not a ligand, but an indirect regulator of IGF-1 bioavailability. Therefore, we speculate that its role in HF disease progression, adding to the deleterious effects of GDF15, might be linked to potential influence over the GH-IGF1 axis. The main function of serum TfR1 is iron uptake, but it can also control sensitivity to erythropoietin in erythroid cells [59]. The only study to date linking TfR1 and HF indicated that the expression of this factor (mRNA levels from myocardial tissue) was reduced in subjects with HF compared with healthy controls [52]. In addition, the same study described how TfR1 was downregulated by both catecholamines and aldosterone in isolated cardiomyocytes. Together with our findings connecting TfR1 and peripheral and central components related to the failing heart, such data highlight the need for future research to determine the role and prognostic validity of this factor in patients with HF.

Galectin-4 (Gal-4) levels were higher in patients with HF vs controls, they covaried with the three components of analysis employed, and carried prognostic value. To our knowledge, only one previous study has put forward a potential link between circulating Gal-4 and HF [13] and the present study is the first to report it in relation to measures of physical capacity and activity. Gal-4 has been studied in relation to cancer and intestinal inflammation [19]. Serum levels of Gal-4 are higher in patients with cancer vs controls, and this difference seems to be greater in those with metastasis [9, 17]. In addition, conflicting roles of Gal-4 (pro- vs anti-inflammatory activity) have been described in different models of inflammatory bowel disease [35, 62]. While it is premature to speculate about the role of circulating Gal-4 in HF, this factor might be linked to some kind of general stress-like stimulus that could be shared across several diseases. However, the specific role of Gal-4 in each disease should be investigated further.

### Study limitations

Despite the effort made to design and conduct an integrative investigation analyzing factors related to function and prognosis of HF, this study had some limitations that need to be acknowledged. In contrast to other studies with nontargeted approaches, the analytical strategy used here did not identify possible pathophysiological and prognostic markers that are unrelated to physical capacity, physical activity, and echocardiographic estimates of disease severity. Moreover, the samples analyzed in this study originated from a homogeneous cohort of elderly patients of European origin with severe HF from a single center, which might limit the external validity of the results. In addition, several potentially confounding features differed between the patient and the control group. Because the proximity extension assay used here to quantify the biomarkers does not allow an absolute quantification of the target proteins, translation into clinically relevant cutoff values is not possible. In addition, mainly because of the availability and quality of antibodies, there are several biomarkers associated with cardiovascular disease or inflammation that are not incorporated in the current multiplex PEA chip. Because the PEA is a form of widespan-targeted analysis, consisting of a curated panel of factors selected for potential relevance in the context of cardiovascular disease, bias is introduced into the analysis, in which inflammatory factors and markers known to be sensitive to cardiac stretch are highly enriched. Therefore, the networks generated could be biased toward these biological functions. Notwithstanding these limitations, we believe that our investigation contributes new understanding of the molecular networks linking the failing heart and the loss of peripheral function in patients with HF.

## Conclusions

This study adds novel and important information about circulatory factors that might link the failing myocardium to the loss of peripheral function. We were able to identify several factors that likely participate in the central–peripheral pathogenesis of HF disease, using a systems biology approach relating echocardiographic assessment of myocardial function, physical capacity, and daily physical activity with the concentrations of circulatory factors that differed in patients with HF vs controls, and Cox regression analysis of mortality. Some of these factors are well-known, validated biomarkers for HF (NT-proBNP, TNF-alpha, and ST2), while others are either relatively or completely new in this field (GDF15, TfR1, and Gal-4). Overall, these findings support the importance of systemic circulatory factors linked to hemodynamic systemic stress and inflammatory responses in the pathogenesis and progress of HF disease.

## Electronic supplementary material

Below is the link to the electronic supplementary material.

Supplementary file1 (DOCX 14 kb)
