# Supplementary material for: Circulatory factors associated with function and prognosis in patients with severe heart failure
Source: Clin Res Cardiol. 2019 Sep 27;109(6):655–72. doi: 10.1007/s00392-019-01554-3 (PMC7239817; doi:10.1007/s00392-019-01554-3)
Supplement: Supplementary file 1 — Supplementary file1 (DOCX 14 kb) [file 392_2019_1554_MOESM1_ESM.docx]

| **Baseline characteristics** | Study group n=66 | All patients n=1467 |
| --- | --- | --- |
| Age (years) | 70 +/-7 | 69.2 +/- 14.0 |
| Female (%) | 20 | 37 |
| NYHA class I / II / III / IV (%) | 0/0/95/5 | 10.4 / 50.6 / 35.1 / 4.0 |
| LVEF (%) |  |  |
| > 50 % | 0 | 21.7 |
| 40 - 49 % | 0 | 25.7 |
| 30 – 39 % | 35 | 20.3 |
| < 30 % | 65 | 32.3 |
| Heart rate (BPM) | 72 +/-8 | 73 +/- 16 |
| Sodium (mmol/l) | 138 +/- 4 | 140 +/- 3 |
| Hemoglobin (g/l) | 141 +/- 12 | 131 +/- 18 |
| NT-proBNP (ng/l) | 2210 +/-1030 | 4124 +/- 6214 |
| Body mass index (kg/m2) | 27.5 +/- 2.5 | 27.0 +/- 5.8 |
| ACEi/ARB (%) | 98 | 83.6 |
| Beta-blockers (%) | 95 | 88.9 |
| Atrial fibrillation (%) | 55 | 53.8 |
| Diabetes (%) | 44 | 28.9 |
| COPD (%) | 17 | 17.8 |
| QRS-duration >120 ms (%) | 20 | 16.2 |
